# Association of BRAF^V600E^ Mutation with the Aggressive Behavior of Papillary Thyroid Microcarcinoma: A Meta-Analysis of 33 Studies

**DOI:** 10.3390/ijms232415626

**Published:** 2022-12-09

**Authors:** Abdallah S. Attia, Mohammad Hussein, Peter P. Issa, Ahmad Elnahla, Ashraf Farhoud, Brandon M. Magazine, Mohanad R. Youssef, Mohamed Aboueisha, Mohamed Shama, Eman Toraih, Emad Kandil

**Affiliations:** 1 Department of Surgery, School of Medicine, Tulane University, New Orleans, LA 70112, USA; 2 School of Medicine, Louisiana State University Health Sciences Center, New Orleans, LA 70112, USA; 3 Genetics Unit, Department of Histology and Cell Biology, Faculty of Medicine, Suez Canal University, Ismailia 41522, Egypt

**Keywords:** BRAF, thyroid cancer, thyroid, microcarcinoma, PTMC

## Abstract

An association between the BRAF^V600E^ mutation and the clinicopathological progression of papillary thyroid microcarcinoma (PTMC) has been suggested. We aimed to summarize the relevant literature and determine the predictive value of BRAF^V600E^ mutation in predicting clinical outcomes and risk stratification in patients with PTMC. A systematic search using PubMed, Cochrane, and Embase up to February 2020 was performed. A total of 33 studies met the inclusion criteria, resulting in a pool of 8838 patients, of whom 5043 (57.1%) patients were positive for BRAF^V600E^ mutation. Tumors with positive BRAF^V600E^ mutation had a higher tendency for multifocality (RR = 1.09, 95%CI = 1.03–1.16), extrathyroidal extension (RR = 1.79, 95%CI = 1.37–2.32), and lymph node metastasis (RR = 1.43, 95%CI = 1.19–1.71). Patients with BRAF^V600E^ mutation were at increased risk of disease recurrence (RR = 1.90, 95%CI = 1.43–2.53). PTMC in patients positive for the BRAF^V600E^ mutation is more aggressive than wild-type BRAF PTMC. Since BRAF-mutated PTMC is generally more resistant to radioiodine treatment, patients with BRAF^V600E^-mutated PTMC may require earlier management, such as a minimally invasive ablative intervention. Conservative management by active surveillance may be suitable for patients with wild-type BRAF^V600E^ PTMC.

## 1. Introduction

Papillary thyroid cancer (PTC) is the most common endocrine malignancy, with detection rates consistently increasing over the past four decades [1]. PTC is generally considered an indolent disease with patients experiencing an acceptable prognosis. Since tumor size is a known risk factor of PTC progression [2], it has been suggested that patients with papillary thyroid microcarcinoma (PTMC), defined as tumors less than or equal to 1.0 cm in diameter, have a better prognosis and may undergo less aggressive treatment. Considering the rather stable incidence-based mortality rates but increased incidence rates of detection, the American Thyroid Association (ATA) supports hemithyroidectomy and active surveillance as potential management options for patients with PTMC [3]. Since early detection and treatment is a long-standing notion in the field of oncology and delayed surgical intervention may increase mortality risk by as much as 94% in thyroid cancer patients [4], it is especially important to determine the tumor molecular characteristics that may predict clinical progression.

The BRAF^V600E^ mutation is caused by substituting valine (V) with glutamic acid (E) at amino acid 600. BRAF mutation is common in patients with PTC, with prevalence rates as high as 51% [5]. BRAF mutation in thyroid cancer occurs only in PTC and PTC-derived anaplastic thyroid cancers, suggesting a role between the two [6]. Mutation of the BRAF oncogene has been associated with extrathyroidal invasion (ETE), lymph node metastasis (LNM), and decreased 10-year survival [6,7,8]. Despite this, a recent 2020 meta-analysis reporting on 11 studies (4674 patients) found that disease recurrence rates were similar between PTC patients with and without BRAF mutation (HR 1.16, 95%CI 0.78–1.71) [9].

Elucidating the clinical implications of BRAF^V600E^ mutation will allow the thyroidology community to better optimize the extent of treatment that patients with PTMC may receive. This meta-analysis aimed to determine the prognostic value of BRAF^V600E^ mutation in predicting clinical outcomes to allow better risk stratification in patients with PTMC.

## 2. Results

### 2.1. Literature Search

**Figure 1:**
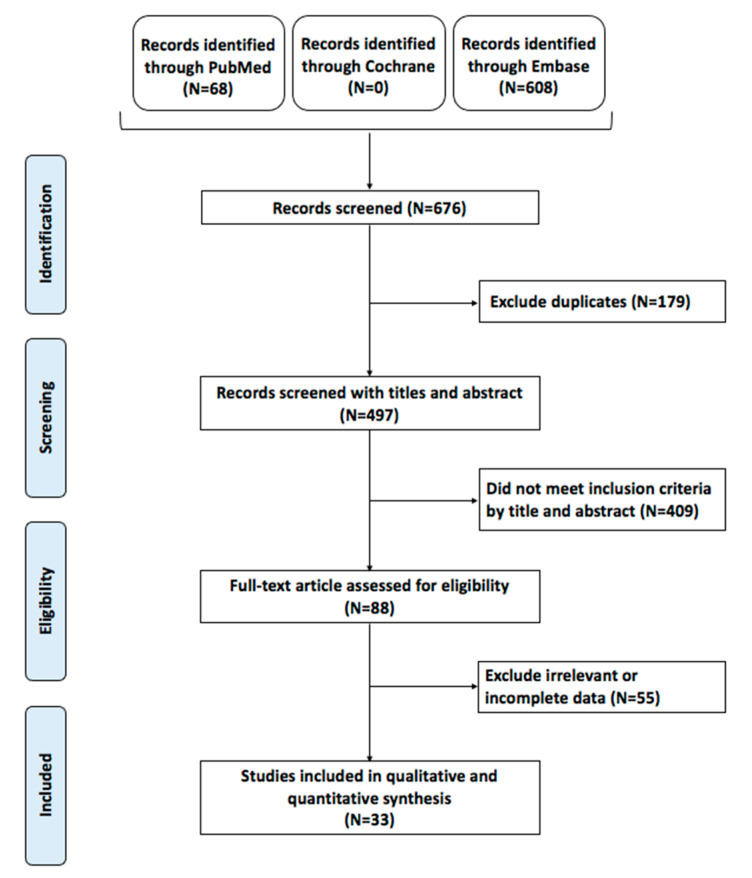
Preferred reporting items for systematic reviews and meta-analyses (PRISMA) flow chart of the included studies.

A total of 676 articles were obtained from the search query, of which 497 remained following duplicate-article deletion. Of these, 409 articles did not meet the initial inclusion criteria, and a total of 88 articles underwent full-text screening. Ultimately, 33 unique articles met the inclusion criteria and were included in this meta-analysis (Figure 1). The works were published between 2005 and 2021, with 11 articles being published within the last 5 years, suggesting interest in the role of BRAF mutation. The articles displayed broad geographic variability, including 8 from China, 8 from South Korea, and 7 from Italy.

### 2.2. Study Population

A total of 8838 patients were included in this meta-analysis. Of these, 5043 (57.1%) patients were positive for the BRAF^V600E^ mutation. Testing for BRAF^V600E^ gene mutation was typically done postoperatively on surgical pathology, while a few studies preoperatively confirmed mutation by fine needle aspiration (FNA) genetic analysis. Characteristics of all 33 included studies are shown in Table 1.

### 2.3. Demographic Characteristics

**Figure 2:**
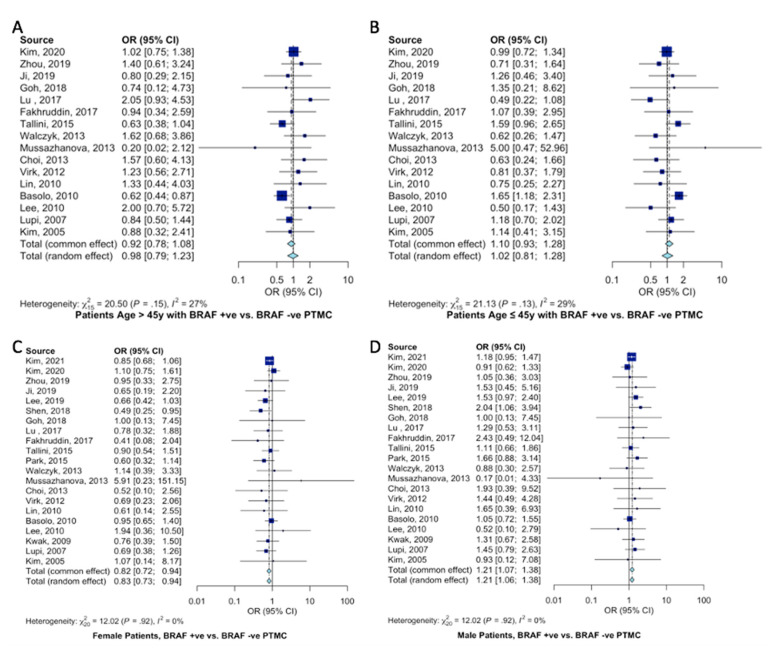
Meta-analysis of demographic characteristics and association with BRAF^V600E^ mutation. Forest plot for (**A**) older patients (>45 years) [11,12,15,19,20,21,22,26,27,29,30,34,35,36,41,42], (**B**) younger patients (≤45 years) [11,12,15,19,20,21,22,26,27,29,30,34,35,36,41,42], (**C**) female patients [10,11,12,15,16,18,19,20,21,22,23,26,27,29,30,34,35,36,37,41,42], and (**D**) male patients [10,11,12,15,16,18,19,20,21,22,23,26,27,29,30,34,35,36,37,41,42].

Overall, 16 studies classified their patient population into young (≤45 years of age) and older (>45 years) cohorts, accounting for 2804 patients. The AJCC recommends a cutoff of 45 years of age. There was no association between a younger (≤45 years of age) patient population and BRAF^V600E^ mutation (OR = 0.92, 95%CI = 0.78–1.08, Figure 2A). Similarly, no association was found in patients older than 45 years of age and BRAF^V600E^ mutation (OR = 1.10, 95%CI = 0.93–1.28, Figure 2B). BRAF^V600E^ mutation was slightly more likely to present in females (OR = 0.82, 95%CI = 0.72–0.94, Figure 2C) than males (OR = 1.21, 95%CI = 1.07–1.38, Figure 2D).

### 2.4. Pathological Features

**Figure 3:**
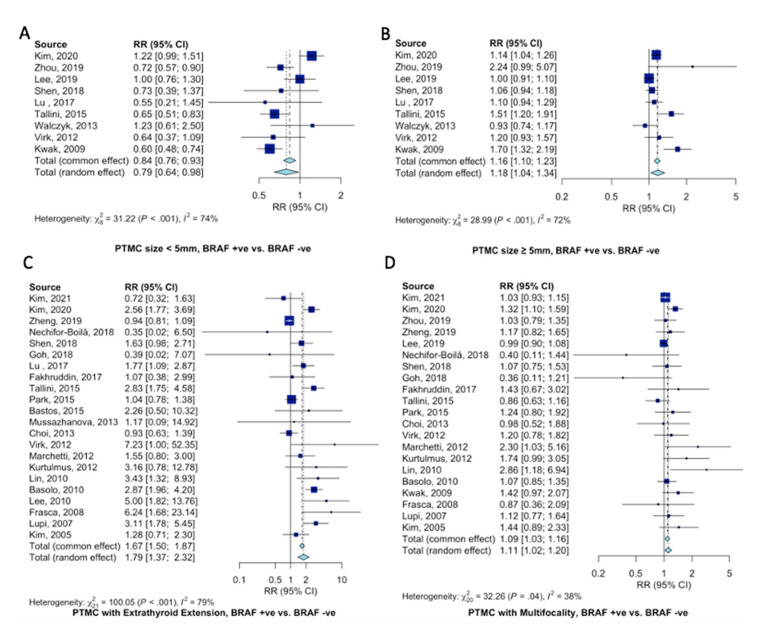
Pooled analysis of clinicopathological characteristics in PTMC patients according to BRAF^V600E^ gene mutation. Forest plots for (**A**) PTMC < 5mm [11,12,16,18,20,22,26,30,37], (**B**) PTMC ≥ 5mm [11,12,16,18,20,22,26,30,37], (**C**) extrathyroidal extension [10,11,13,17,18,19,20,21,22,23,24,27,29,30,32,33,34,35,36,39,41,42], and (**D**) multifocality [10,11,12,13,16,17,18,19,21,22,23,29,30,32,33,34,35,37,39,41,42].

Pooled estimates of pathological and clinical characteristics of PTMC patients according to the BRAF^V600E^ gene mutation were analyzed. Tumors with positive BRAF^V600E^ mutation were less likely to be <5 mm in size (RR = 0.79, 95%CI = 0.64–0.98, Figure 3A) and at increased odds of being ≥5mm in size (RR = 1.18, 95%CI = 1.04–1.34, Figure 3B). BRAF^V600E^ mutant PTMCs were at 79% increased risk of displaying extrathyroidal extension (RR = 1.79, 95%CI = 1.37–2.32, Figure 3C) and 9% increased risk of displaying tumor multifocality (RR = 1.09, 95%CI = 1.03–1.16, Figure 3D).

**Figure 4:**
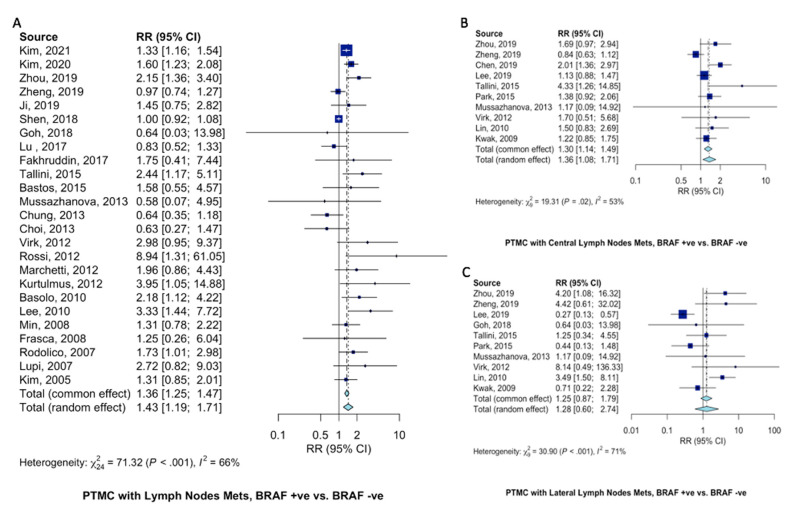
Pooled analysis of lymph node metastasis in PTMC patients according to BRAF^V600E^ mutation. Forest plots for (**A**) LNM [10,11,12,13,15,18,19,20,21,22,24,27,28,29,30,31,32,33,35,36,38,39,40,41,42], (**B**) central LNM [12,13,14,16,22,23,27,30,34,37], and (**C**) lateral LNM [12,13,16,19,22,23,27,30,34,37].

Compared to patients with wild-type BRAF^V600E^ PTMC, patients with BRAF^V600E^-mutant PTMC were at 43% increased risk of presenting with LNM (RR = 1.43, 95%CI = 1.19–1.71, Figure 4A). Specifically, BRAF^V600E^ mutation increased the risk of central lymph node metastasis 36% (RR = 1.36, 95%CI = 1.08–1.71, Figure 4B). Seemingly, since PTMC typically spread through the central compartment, there only tended to be an increase in lateral LNM (RR = 1.28, 95%CI = 0.60–2.74, Figure 4C).

**Figure 5:**
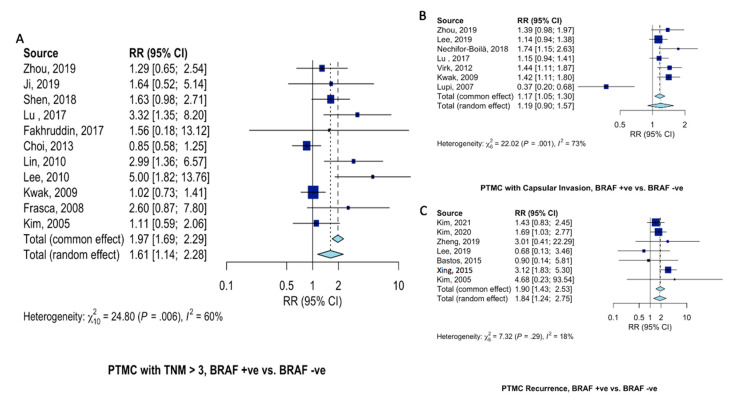
Pooled analysis of clinical characteristics in PTMC patients according to BRAF^V600E^ gene mutation. Forest plots for (**A**) advanced (TNM > 3) disease stage [12,15,18,20,21,29,34,36,37,39,42], (**B**) capsular invasion [12,16,17,20,30,37,41], and (**C**) recurrence [10,11,13,16,24,25,42].

Patients with BRAF^V600E^-mutant PTMC at 61% (RR = 1.61, 95%CI = 1.14–2.28, Figure 5A) increased risk of presenting with advanced (TNM > 3) clinical stage. Risk of capsular invasion were similar (RR = 1.19, 95%CI = 0.90–1.57, Figure 5B) between PTMC patients with and without BRAF^V600E^ mutation. Disease recurrence was almost twice as likely in patients with BRAF^V600E^-mutant PTMC (RR = 1.90, 95%CI = 1.43–2.53, Figure 5C) than those not harboring BRAF^V600E^ mutation.

## 3. Discussion

PTMC is the most common malignant tumor in patients aged 45 years and older [43]. Though most patients display excellent prognosis, a recent work of ours using the National Cancer Database (N = 5628 patients) found that 19% of patients with PTMC present with advanced features (defined as lymph node metastasis, extrathyroidal extension, or lymphovascular invasion) [44]. In around 45% of PTC, BRAF mutation is present [6]. Our results demonstrate that the BRAF^V600E^ mutation was associated with larger PTMC’s (≥5mm but <10mm), multifocality, ETE, advanced stage, and higher recurrence rates. Considering that PTMC with BRAF mutation may not be as indolent a disease as conventionally thought, our work may assist surgeons and endocrinologists in appropriate treatment planning.

BRAF^V600E^ mutation is prevalent in approximately 45% in PTC, though this value drops to around 30% in TNM I and II PTC [6]. Therefore, approximately one in three PTMC cases involve BRAF mutation [45]. This is consistent with our study findings, which found a significant relationship between tumor size ≥ 5mm and the BRAF^V600E^ mutation. Accordingly, disease progression is known to correlate with tumor size [11], such that PTMC tumors ≥ 5 mm more often present with central lymph node metastasis than those < 5mm [46,47,48]. Therefore, patients with PTMC ≥ 5mm with confirmed BRAF mutation should be appropriately counseled and closely monitored for disease progression.

Numerous works have found BRAF^V600E^ mutation to be associated with a worse initial presentation [49,50]. Our results found that tumors with BRAF^V600E^ mutation were almost twice as likely (RR = 1.79) as those with BRAF wild-type PTMC to develop ETE. Interestingly, a recent 2020 work by Tallini et al. reported that PTMCs > 5mm in size were more frequently located in the peripheral region of the thyroid or were “subcapsular” [51]. The authors hypothesized that these tumors were influenced by the exterior microenvironment. Peripherally located PTMC has been associated with infiltrative growth, lymph node metastasis, and BRAF^V600E^ mutation [30,52,53]. Our results also found that BRAF^V600E^ mutated PTMC were approximately 40% more likely to develop lymph-node metastasis (RR = 1.43). This is consistent with several primary studies. Since the presence of clinicopathologic features such as LNM and ETE in BRAF-mutated PTMC allow synergistic aggressive behavior [8,25], patients with advanced PTMC disease are appropriately classified as “intermediate risk” as opposed to “low risk” by current ATA guideline recommendations [3].

Since patients with PTMC have an extremely low mortality rate, the bulk of patient clinical management lies in preventing and identifying disease recurrence [45]. Our analysis found that patients with BRAF-mutated PTMC are almost twice as likely as those with wild-type BRAF PTMC to develop disease recurrence (RR = 1.90). These findings are consistent with a recent multicenter international study that included 742 patients, which found that overall disease recurrence was 6.4% (32/502) in wild-type BRAF tumors but increased significantly to 10.8% (26/241) in BRAF-mutated PTC (*p* = 0.041, [11]). On multivariate analysis of low-risk PTMC, the authors found that BRAF mutation conferred six times the chance of disease recurrence (HR = 6.65, 95%CI = 1.80–24.65, [11]). Considering a reported negative predictive value of BRAF mutation of 98.7% with respect to disease recurrence in low-risk PTMC [11], BRAF mutation identification should be considered in patients seeking management by conservative methods such as active surveillance. 

The management of PTMC has been long-debated, with the most recent ATA guidelines allowing consideration of active surveillance in an attempt to prevent overly aggressive intervention [3]. Active surveillance is the close monitoring of patients with PTC or PTMC with routine imaging screening for disease progression [54,55]. The potential for complications during thyroidectomy, such as recurrent laryngeal nerve paresis and hypoparathyroidism, make active surveillance an attractive treatment option (). The current ATA guidelines recommend surgery for patients with primary thyroid cancers but recommend considering conservative active surveillance in patients with very low-risk tumors (e.g., no clinical evidence of disease) or who are at high surgical risk (e.g., worrisome comorbid conditions). The guidelines recommended future studies to elucidate the role of BRAF mutation before its incorporation into risk stratification [3]. Considering the accordingly increased risk of tumor multifocality, ETE, capsular invasion, lymph node metastasis, and disease recurrence in BRAF mutated PTMC, patients with such tumors may be appropriately recommended an intervention of moderate aggressiveness, such as minimally invasive ablative techniques. Radiofrequency ablation (RFA) is one treatment option that has demonstrated impressive safety and efficacy profiles [56,57]. A 2021 study of 102 patients with PTMC found 100% resorption rates at the 5-year follow-up [58]. Befittingly, minimally invasive ablative technology is generally more efficacious in nodules of smaller sizes [59,60], making them an attractive option for patients with PTMC. Additionally, since BRAF mutation is thought to silence thyroid iodide-handling genes and make these carcinomas more resistant to radioiodine treatment, surgeons and endocrinologists should consider immediate management [6].

Our study is not without limitations. First, though the large sample size allowed for powerful analysis, all studies included a retrospective study design. The authors acknowledge this limitation and attempted to address it by evaluating the degree of bias. Additionally, while the studies represent a breadth of populations and allow for greater data generalizability, sub-group analyses for patient race were not possible as they were frequently not reported.

## 4. Materials and Methodology

### 4.1. Literature Search

This meta-analysis was conducted in accordance with the Preferred Reporting Items for Systematic Review and Meta-Analyses (PRISMA) guidelines. Three databases, including PubMed, Cochrane Library, and Embase were searched for primary peer-reviewed articles through November 2022. The following search terms were used: (thyroid) AND (microcarcinoma) AND (BRAF^V600E^). Reference lists from relevant review articles and included studies were also searched. Only articles reported in the English language were considered for inclusion. All articles that met the following inclusion criteria were considered for inclusion: studies that (a) were randomized controlled trials, observational design studies including cross-sectional, case-control, and/or cohort designs, (b) described PTMC, (c) considered patients with BRAF^V600E^ mutation, and (d) analyzed potential prognostic factors in patients. Articles were excluded if they met the following criteria: (a) were review papers, conference papers, editorial letters, case reports, abstracts, or comments, (b) not reported in the English language, (c) did not report on PTMC patients with BRAF^V600E^ mutation.

Results of the search query were screened for inclusion by two independent authors (A.S.A., A.E.), who screened first by title and abstract, and subsequently by full-text eligibility. A total of 33 articles met the final criteria, and their data were independently extracted into a pre-designed data sheet. Variables collected included the study first author, year of publication, the country where the study took place, study design, total sample size, age, gender, as well as clinicopathological features including BRAF^V600E^ mutation status, tumor multifocality, LNM, ETE, tumor stage. Tumor staging included advanced stage >T2, recurrence rates, and tumor size with a cutoff value of 5mm where large tumors meant that the tumor was ≥ 5mm but less than 10mm. Discrepancies in screening or extraction were resolved by re-examination of the relevant study until consensus was achieved. Disagreements were resolved by discussion with a senior author.

### 4.2. Data Analysis

Data were analyzed using RStudio version 4.2.2 (meta and metafor package) (citation packages). Dichotomous values were used as input. Z-score (one-tail) at the optimum cutoff value was calculated if the data were reported as mean and standard deviation and the equivalent percentage of patients above and below the threshold was calculated. The Mantel–Haenszel method [61,62] was employed to calculate the common effect estimate and between-study heterogeneity statistic Q using the DerSimonian–Laird estimator [63]. Data were presented as risk ratio (RR) or odds ratio (OR) along with a 95% confidence interval (CI). Heterogeneity was examined by the chi-squared Q test and I2 statistic. The fixed or random-effects model was applied according to the presence or absence of heterogeneity. Publication bias was assessed using a funnel plot for precision and Egger’s regression test (Table S1 and Figure S1).

## 5. Conclusions

PTMC positive for the BRAF^V600E^ mutation is more aggressive than wild-type BRAF PTMC. Since BRAF-mutated PTMC is generally more resistant to radioiodine treatment, patients with BRAF^V600E^-mutated PTMC may require earlier management, such as a minimally invasive ablative intervention. Conservative management by active surveillance may be suitable for patients with wild-type BRAF^V600E^ PTMC.

## Figures and Tables

**Table 1:** Characteristics of the articles included.

Author	Year	Country	Study Design	PTMC Total	% BRAF Mutated
Kim [10]	2021	South Korea	Retro	2319	60.16
Kim [11]	2020	Multicenter study	Retro	743	32.44
Zhou [12]	2019	China	Retro	162	83.33
Zheng [13]	2019	China	Retro	299	83.28
Chen [14]	2019	China	Retro	182	47.80
Ji [15]	2019	China	Retro	89	75.28
Lee [16]	2019	South Korea	Retro	911	78.70
Nechifor-Boilă [17]	2018	Romania	Retro	25	36.00
Shen [18]	2018	China	Retro	236	62.29
Goh [19]	2018	Singapore	Retro	21	33.33
Lu [20]	2017	China	Retro	108	54.63
Fakhruddin [21]	2017	Lebanon	Retro	75	72.00
Tallini [22]	2015	Italy	Retro	264	50.00
Park [23]	2015	South Korea	Retro	460	79.78
Bastos [24]	2015	Brazil	Retro	40	52.50
Xing [25]	2015	Multicenter	Retro	534	41.01
Walczyk [26]	2013	Poland	Retro	113	69.03
Mussazhanova [27]	2013	Japan	Retro	13	46.15
Chung [28]	2013	South Korea	Retro	111	22.52
Choi [29]	2013	South Korea	Retro	101	71.29
Virk [30]	2012	US	Retro	124	70.16
Rossi [31]	2012	Italy	Retro	50	68.00
Marchetti [32]	2012	Italy	Retro	85	74.12
Kurtulmus [33]	2012	Turkey	Retro	64	29.69
Lin [34]	2010	China	Retro	61	34.43
Basolo [35]	2010	Italy	Retro	578	39.62
Lee [36]	2010	China	Retro	64	37.50
Kwak [37]	2009	South Korea	Retro	339	62.83
Min [38]	2008	South Korea	Retro	60	53.33
Frasca [39]	2008	Italy	Retro	103	24.27
Rodolic [40]	2007	Italy	Retro	214	41.12
Lupi [41]	2007	Italy	Retro	230	39.13
Kim [42]	2005	South Korea	Retro	60	51.67

Retro: retrospective.

## Data Availability

Data are contained within the article.

## Supplementary Materials

The following supporting information can be downloaded at https://www.mdpi.com/article/10.3390/ijms232415626/s1.
