# Supplementary material for: Association of BRAFV600E Mutation with the Aggressive Behavior of Papillary Thyroid Microcarcinoma: A Meta-Analysis of 33 Studies
Source: Int J Mol Sci. 2022 Dec 9;23(24):15626. doi: 10.3390/ijms232415626 (PMC9779545; doi:10.3390/ijms232415626)
Supplement: Supplementary file 1 [file ijms-23-15626-s001.zip › ijms-2033673-supplementary.pdf]

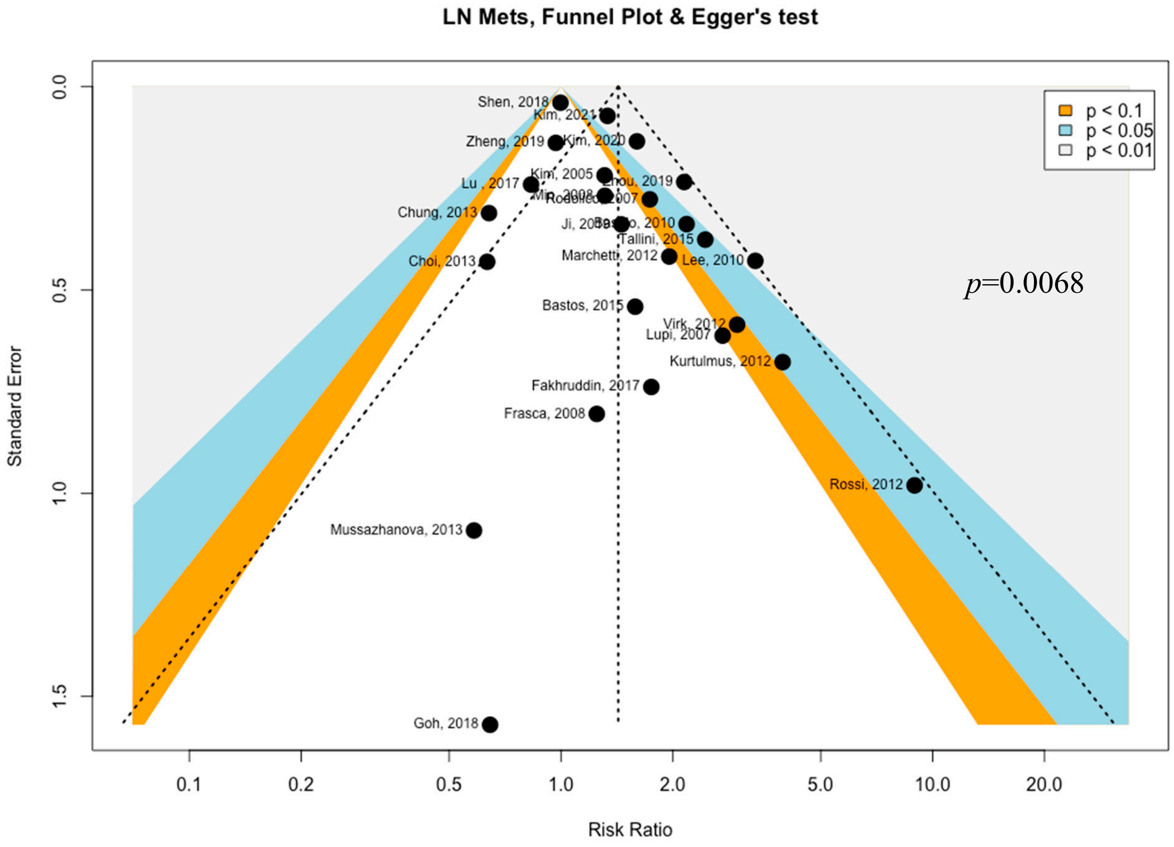

Supplemental Figure S1. Funnel plot for publication bias. Egger's test was performed.

Supplementary Table S1. Pooled analysis for pathological and clinical parameters.

Characteristics	Number studies	Sample size				Effect size			Heterogeneity			Publication bias
		**Total**	**BRAF +**	**BRAF -**	**Model**	**OR**	**95% CI**	***p*-value**	I^2	***p*-value**	**Tau^2**	**P (Egger's test)**
**Pathological data**												
Multifocal	18	4,048	2,200	1,848	F	1.31	1.13, 1.51	<0.001	21%	0.20	0.027	0.60
Tumor size (>5mm)	9	1,036	642	394	F	1.69	1.88, 2.41	0.004	0.0%	0.89	0.16	0.34
Capsular invasion	4	634	416	218	F	2.15	1.49, 3.09	<0.001	0.0%	0.56	0.0	0.11
ETE	20	3,772	1,962	1,810	R	2.38	1.71, 3.30	<0.001	61.7%	<0.001	0.27	0.90
Overall LNM	26	4,867	2,567	2,300	R	1.58	1.02, 2.42	0.037	52.3%	0.001	0.84	0.47
Central LNM	10	1,943	1,313	630	R	1.62	1.03, 2.52	0.034	48.4%	0.042	0.87	0.27
Lateral LNM	9	1,761	1,226	535	R	1.97	0.96, 4.02	0.06	53.5%	0.028	0.55	0.28
**Clinical data**												
Advanced stage	10	1,607	926	681	R	1.60	1.22, 2.11	0.001	59.8%	0.008	0.32	0.08
Recurrence	4	534	219	315	F	2.49	1.69, 3.68	**<0.001**	5.9%	0.36	0.01	0.54

Pooled analysis was calculated using Mantel-Haenszel and measured as odds ratio (OR) and 95% confidence interval (CI). R: Random-effects model, F: Fixed- effects model, ETE: extrathyroid extension, LNM: lymph node metastasis (LNM). I^2: the ratio of true heterogeneity to total observed variation.
